# Handbook of Secure Care

**DOI:** 10.1192/pb.bp.115.052704

**Published:** 2017-12

**Authors:** Lindsay Thomson

The *Handbook of Secure Care* is a useful book for those new to the field of forensic mental health and is most relevant to those practising in England and Wales. It examines the relationship between mental disorder and offending, with individual chapters on personality disorder, intellectual disability, autism spectrum disorder and acquired brain injury. Strangely, there is little on psychosis which is the fundamental diagnosis within secure care.

The work considers the needs of specific populations such as women, young and older people, and outlines the provision of secure psychiatric services for these groups. It focuses on the basic components of secure care and includes information on risk assessment and management, and on recovery. The latter chapter is of particular use in defining the challenges we face in secure care and ways to redefine our conventional thinking. The fundamentals of psychological treatment in secure care are clearly set out and there is a helpful description of the role of nursing within that setting.

There is discussion in the first chapter on the evolution of secure and forensic mental healthcare, as well as information on the number of secure beds, but I would have welcomed an analysis of the overall estate, the needs for planning and the methods of provision. Similarly, details on pathways into or out of secure care, or on the legislation that allows us to detain people within these settings would have been valuable.

Notably, there is a good chapter by Penny & Exworthy on human rights in secure psychiatric care – the Human Rights Act 1998 underpins much of what we do in secure care, making this especially relevant. It is followed by a chapter on quality assurance and clinical audit. It is my view that the human rights considerations and the quality improvement agenda are so crucial to our work that it would have been beneficial to place these chapters near the beginning of the book to emphasise their importance.

